# Participant recruitment into a randomised controlled trial of exercise therapy for people with multiple sclerosis

**DOI:** 10.1186/s13063-015-0996-3

**Published:** 2015-10-15

**Authors:** Anouska Carter, Liam Humphreys, Nicky Snowdon, Basil Sharrack, Amanda Daley, Jane Petty, Nicola Woodroofe, John Saxton

**Affiliations:** The Centre for Sport and Exercise Science, Health and Wellbeing Research Institute, Sheffield Hallam University, Collegiate Hall, Collegiate Crescent, Sheffield, S10 2BP UK; Academic Department of Neuroscience, Sheffield Teaching Hospitals NHS Foundation Trust, Sheffield, UK; Primary Care Clinical Sciences, University of Birmingham, Birmingham, UK; Multiple Sclerosis Society, London, UK; Biomedical Research Centre, Sheffield Hallam University, Sheffield, UK; Department of Sport, Exercise and Rehabilitation, Northumbria University, Northumbria, UK

**Keywords:** Exercise, Multiple sclerosis, Randomised controlled trials, Recruitment

## Abstract

**Background:**

The success of a clinical trial is often dependant on whether recruitment targets can be met in the required time frame. Despite an increase in research into the benefits of exercise in people with multiple sclerosis (PwMS), no trial has reported detailed data on effective recruitment strategies for large-scale randomised controlled trials. The main purpose of this report is to provide a detailed outline of recruitment strategies, rates and estimated costs in the Exercise Intervention for Multiple Sclerosis (ExIMS) trial to identify best practices for future trials involving multiple sclerosis (MS) patient recruitment.

**Methods:**

The ExIMS researchers recruited 120 PwMS to participate in a 12-week exercise intervention. Participants were randomly allocated to either exercise or usual-care control groups. Participants were sedentary, aged 18–65 years and had Expanded Disability Status Scale scores of 1.0–6.5. Recruitment strategies included attendance at MS outpatient clinics, consultant mail-out and trial awareness-raising activities.

**Results:**

A total of 120 participants were recruited over the course of 34 months. To achieve this target, 369 potentially eligible and interested participants were identified. A total of 60 % of participants were recruited via MS clinics, 29.2 % from consultant mail-outs and 10.8 % through trial awareness. The randomisation yields were 33.2 %, 31.0 % and 68.4 % for MS clinic, consultant mail-outs and trial awareness strategies, respectively. The main reason for ineligibility was being too active (69.2 %), whilst for eligible participants the most common reason for non-participation was the need to travel to the study site (15.8 %). Recruitment via consultant mail-out was the most cost-effective strategy, with MS clinics being the most time-consuming and most costly.

**Conclusions:**

To reach recruitment targets in a timely fashion, a variety of methods were employed. Although consultant mail-outs were the most cost-effective recruitment strategy, use of this method alone would not have allowed us to obtain the predetermined number of participants in the required time period, thus leading to costly extensions of the project or failure to reach the number of participants required for sufficient statistical power. Thus, a multifaceted approach to recruitment is recommended for future trials.

**Trial registration:**

International Standard Randomised Controlled Trial Registry number: ISRCTN41541516; date registered: 5 February 2009.

**Electronic supplementary material:**

The online version of this article (doi:10.1186/s13063-015-0996-3) contains supplementary material, which is available to authorized users.

## Background

One of the most difficult challenges in clinical trials is whether appropriate participants can be identified and consented quickly [1]. Many trials either fail to reach recruitment targets or have to be extended [2]. This leads to either an underpowered study or an extension of the duration of the study, often at additional cost, impacting the time required to inform clinical practice and using funds that could have been used for other research [3, 4]. The implementation of an efficient and effective recruitment strategy for patients in clinical trials is critical if expensive delays and failure to meet predetermined targets are to be avoided [5].

The introduction of Consolidated Standards of Reporting Trials (CONSORT) guidelines [6] has improved the quality of recruitment information reported for randomised controlled trials (RCTs). However, detailed data on recruitment, including methods used, rates achieved and cost are still underreported. More detailed data would help to identify strategies to improve recruitment, benefiting both researchers and research [4] and ultimately patients.

In recent years, there has been an increase in the number of studies of the possible health benefits of exercise for people with multiple sclerosis (PwMS) [7–9]. Although detailed recruitment data for exercise interventions in other clinical populations, such as patients with breast cancer and wheelchair users, are available [10–12], to date no study has reported recruitment data for a large-scale RCT of exercise for PwMS. In recent years, the number of clinical trials in multiple sclerosis (MS) has increased, leading to an increased need to recruit research participants from a limited patient pool, and with modern trials often needing large sample sizes to ensure adequate statistical power [13].

The Exercise Intervention for Multiple Sclerosis (ExIMS) trial was a large-scale RCT involving 120 people with mild to moderate MS. It was designed to investigate the short- and longer-term health impacts of a 12-week pragmatic exercise programme [14, 15]. The main purpose of this report is to provide a detailed outline of the recruitment methods, rates and estimated cost to help inform future research of this type. In addition, we aim to determine which recruitment method provided the highest yield of participants and the lowest cost per participant.

## Methods

### Trial design

Only a brief description of the trial design is reported here, as detailed protocol and outcomes papers for this study have been published elsewhere [14–16]. Power calculations indicated that we would need 100 PwMS to complete the trial. This, alongside the retention rates observed in our feasibility study of 87 % immediately following the intervention and 80 % at 3 months [17], led to a recruitment target of 120 PwMS (60 in each group). The project was funded for 3 years, and an initial recruitment target of five participants per month over 24 months was set, with recruitment beginning in February 2009. A sample of 120 PwMS with mild to moderate disability [Expanded Disability Status Scale (EDSS) score ≤6.5] was recruited. Participants were randomized to a 12-week pragmatic exercise intervention (2 × supervised and 1 × home-based session per week for 6 weeks, followed by 1 × supervised and 2 × home-based sessions per week for 6 weeks, plus usual care) or usual care alone. The primary outcome was self-reported exercise behaviour at 3 months using the Godin Leisure Time Exercise Questionnaire [18]. In addition, accelerometry was used to provide an objective measure of daily activity and step count (ActiGraph GT2M accelerometer; ActiGraph, Pensacola, FL, USA). Secondary outcome measures included fatigue, health-related quality of life, functional ability and neurological impairment. Outcomes were assessed at baseline, immediately postintervention (3 months) and 6 months postintervention (9 months). This study was approved by the South Yorkshire Research Ethics Committee (08/H1310/69) according to the principles of the Declaration of Helsinki, and all participants provided informed consent before enrolment.

### Eligibility criteria

Regardless of the recruitment method used, all participants were screened by a consultant neurologist before entering the trial. Participants were included if they (1) had a clinical diagnosis of MS based on the McDonald diagnostic criteria for MS [19], (2) had an EDSS score [20] between 1.0 and 6.5, (3) were between 18 and 65 years of age, (4) were stable on disease modifying treatment for at least 3 months before recruitment, (5) were clinically stable (had not experienced a relapse in at least 4 weeks), (6) were physically able to participate in exercise three times per week and (7) were able to provide written informed consent. The exclusion criteria were (1) failure to meet any of the inclusion criteria, (2) experiencing illness that would be a contraindication to exercise, (3) living farther than 20 miles from the trial centre, (4) unwilling to be randomised to either group and (5) already engaged in moderate structured exercise at least three times per week for at least 30 minutes per session consistently for the last 6 months. Participants who were initially screened out either for having changed their drug treatment in the last 3 months or for having had a relapse in the previous 4 weeks were reassessed following the required lapse of time and recruited if the eligibility criteria were then met.

### Recruitment methods

Participants were recruited continuously until the required sample size was obtained. All recruitment methods and procedures were approved by the South Yorkshire Research Ethics Committee. Regardless of recruitment method, we adhered to the following procedures (Table 1).

**Table 1:** Recruitment process for Exercise Intervention for Multiple Sclerosis trial

Recruitment process
• Potentially eligible participants identified (consultant neurologist, mail-out, other)
• Trial manager made aware of participants interest
• Trial manager speaks (by telephone or in person) with participant to outline study, answer questions and screen participants for all eligibility criteria
• If interested and eligible, participant booked in for trial familiarisation session (by telephone or in person)
• Potential participant attends trial familiarisation at trial site and is given 7 days to consider participation
• Participant booked in for initial appointment to provide informed consent and participate in baseline assessment

#### Consultant referral at MS outpatient clinic

Consultant referral at MS outpatient clinics was the primary recruitment strategy, as consultant recommendations are thought to play a crucial role in participants’ decisions to enrol in a clinical trial [1]. In addition, recruitment by this method would reduce the possibility of contacting patients who did not meet the eligibility criteria.

MS outpatient clinics took place at the Royal Hallamshire Hospital, Sheffield, UK, on a weekly basis. The project’s lead consultant (BS) and two other neurology consultants assisted with identifying potentially eligible and interested participants. Each consultant saw approximately 13 patients per clinic (10 follow-ups and 3 new patients) over a 3.5-h period. A trial researcher attended all clinics, enabling any participants identified to speak with them about the trial, ask any questions and confirm eligibility. If interested, participants were booked in for a familiarisation session at the trial site.

#### Consultant mail-out

To maintain a consistent flow of patients into the study, participant mail-outs were timed to take place during periods of low recruitment. Letters were sent in batches of no more than 125 to manage the flow of patients into the study and ensure that all participants who responded could be contacted in a timely manner. All mail-outs were personalised and sent by the project’s lead consultant (BS) and contained the logos of the hospital, the university and the funding body (MS Society). The details of the participants to be included in the mail-outs were obtained from the local MS risk-sharing scheme database and clinic waiting lists. Notes of potential participants were screened for all available eligibility criteria (clinical diagnosis of MS, distance from trial centre, EDSS score and age). In addition, those who had been contacted previously about the project through other means and stated that they did not wish to take part were screened out at this stage. Letters contained a reply slip and a stamped, addressed envelope and the participant information sheet, along with a contact number for further information. The trial manager contacted all interested participants upon receipt of the reply slip to answer any questions and confirm eligibility. No attempt was made to contact patients who did not respond to the invitation letter from their consultant.

#### Trial awareness strategies

Other trial awareness strategies included leaflets and posters at clinics, therapy centres and regional MS societies, presentations and attendance at regional MS Society events and to local MS physiotherapy teams, referral from other professionals such as MS nurses and word of mouth. Despite being reported as a potentially successful recruitment method [10], we chose not to use local media (radio, television and newspapers), as it was felt that this might attract too many individuals who would not meet the study eligibility criteria. It was agreed that this strategy would be used only as a last resort.

### Incentives

Participants were reimbursed for travel costs (40 p/mile up to a maximum of £10/visit) for all visits to the trial centre, with free parking made available. Those more severely disabled were also offered the option of using a taxi service if other methods of transport would restrict their ability to participate. Flexible appointment times and start dates were made available to help participants fit the trial commitments around work, child care and fatigue patterns. To encourage participation, the usual-care group was offered up to four exercise sessions following the study. This option was taken up by 20 % of the usual-care participants who completed the study.

### Data analysis

Participant recruitment rates were calculated as the average number of participants recruited per month over the duration of the recruitment period. Response rates were reported as percentage interested and percentage recruited. Recruitment yields were calculated as total recruited divided by the number of interested participants. Recruitment time was estimated on the basis of time taken to ascertain interest and eligibility in the study and did not include any other time taken to carry out familiarisation visits and consent, as this was the same for all recruitment methods. The time cost of each method was calculated per participant recruited, based on the average salary cost per hour of the trial researcher.

## Results

**Fig. 1:**
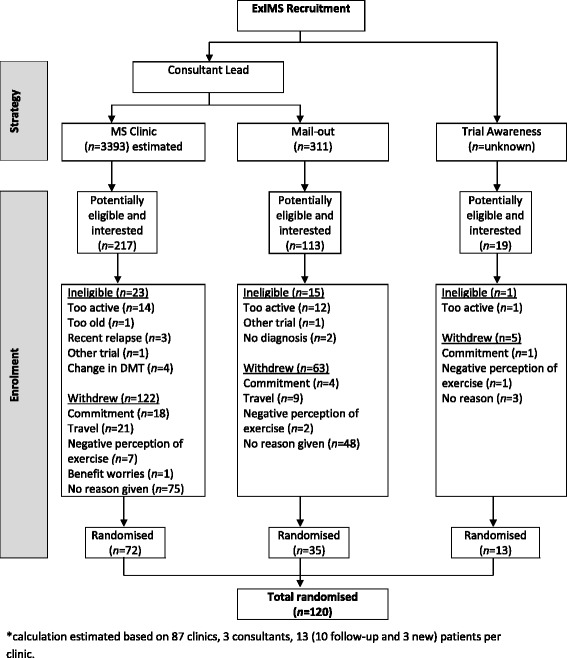
Flow diagram of participant recruitment to the Exercise Intervention for Multiple Sclerosis (ExIMS) trial. *DMT* disease-modifying therapy *MS* multiple sclerosis

A total of 349 potentially eligible participants were identified via the recruitment methods (217 MS clinic, 113 consultant mail-out and 19 trial awareness) (see Fig. 1). For CONSORT checklist and flow diagram please see Additional file 1.

### Recruitment rates

**Fig. 2:**
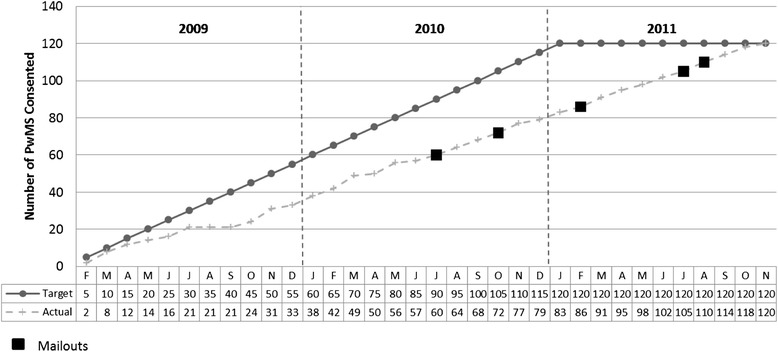
Predicted and actual recruitment rates for participants in the Exercise Intervention for Multiple Sclerosis research trial. *PwMS* people with multiple sclerosis

The original recruitment period was planned to take place over the course of 24 months. This was extended to a period of 34 months (February 2009 to November 2011), owing to a lower than expected recruitment rate of 3.5 ± 0.32 [mean ± 95 % confidence interval (CI)] participants per month (see Fig. 2). Recruitment was carried out by attending MS clinics and using trial awareness strategies throughout this period. Mail-outs were conducted in the second year of the trial at time points where lower levels of recruitment from the clinic were observed in the trial’s first year (July, August, February and October).

### Response rates

Of approximately 3393 people with MS who attended the MS outpatient clinic during the recruitment period, 217 (6.4 %) were identified as potentially interested and eligible. Of these 217, 23 (10.6 %) were ineligible and 122 (56.2 %) declined to participate.

Mail-outs were sent to 311 potentially eligible participants. Of these candidates, 133 (42.8 %) PwMS expressed an interest in the trial, 15 (11.2 %) of whom were ineligible and 63 (47.3 %) of whom declined to participate.

Our trial awareness strategies provided 19 interested individuals from among an unknown pool of potential participants, one (5 %) of whom was ineligible and four (21.1 %) of whom declined to participate.

### Randomisation yields/accrual rates

The randomisation yield was 33.2 % (72 of 217) from the MS clinic, 31.0 % (35 of 113) for consultant mail-outs and 68.4 % (13 of 19) for those contacted via trial awareness strategies. This led to 60 % (72 of 120) of participants being recruited via MS clinics, 29.2 % (35 of 120) via mail-outs and 10.8 % (13 of 120) via trial awareness strategies.

### Reasons for ineligibility

A total of 39 participants (23 MS clinic, 15 mail-out and 1 trial awareness) who had expressed an interest were ineligible. In order of prevalence, the main reasons for ineligibility were too active (27 of 39; 69.2 %), recent change in disease-modifying therapy (4 of 39; 10.3 %), recent MS relapse (3 of 39; 7.7 %), participating in another trial (2 of 39; 5.1 %), no definitive diagnosis of MS (2 of 39; 5.1 %) and too old (1 of 39; 2.6 %).

### Reasons for declining participation

The reasons that eligible participants declined to participate, in order of prevalence, were no reason given (126 of 190; 66.3 %), issues with transport/travel to the trial site (30 of 190; 15.8 %), other commitments (23 of 190; 12.1 %), negative perceptions of exercise (10 of 190; 5.3 %) and loss of benefit worries (1 of 190; 0.5 %).

### Recruitment time/cost

MS clinics required the longest recruitment time of 4.2 h per participant, whilst the consultant mail-out had the shortest recruitment time of 0.6 h per participant (see Table 2).

**Table 2:** Estimated time to identify and recruit participants and the associated costs

Recruitment method	Time spent recruiting (h)	Time per potential participant (h)^a^	Time per recruited participant (h)^b^	Cost per recruit (based on estimated cost of a researcher £25/h)
MS outpatient clinic	304.5 (87 clinics)	1.4 (304.5/217)	4.2 (304.5/72)	£105 (£25 × 4.2)
Consultant mail-out	20 (5 mail-outs)	0.2 (20/113)	0.6 (20/35)	£15 (£25 × 0.6)
Trial awareness strategies	26^c^	1.5 (29/19)	2.2 (29/13)	£55 (£25 × 2.2)
All strategies	350.5	1.4 (350.5/349)	2.9 (350.5/120)	£72.50 (£25 × 2.9)

*MS* multiple sclerosis

^a^Time per recruited participant (h) is calculated as time spent recruiting/number of participants recruited

^b^An estimated 26 h were spent raising awareness of the trial, which included producing a flyer and attending and giving talks at various MS events

^c^Time per potential participant (h) is calculated as time spent recruiting (h)/number of potentially eligible participants

## Discussion

### Recruitment rates

Recruitment into this study was slower than anticipated at 3.5 ± 0.32 (mean ± 95 % CI) participants per month, leading to the trial’s failing to recruit on time and an extended recruitment period of 34 months (from an initial target of 24 months) needed to reach the target number of participants. Recruitment rates have not been reported previously for large-scale exercise trials in PwMS, but investigators who studied a non-exercise intervention using computerised cognitive behavioural therapy for PwMS reported slightly lower rates of 2.6/month [21]. Researchers in a multicentre RCT for a group-based fatigue management programme reported recruitment of 13.0 participants per month (across 3 sites), equating to 4.3 per trial site [22]. However, both these trials had a lower patient time commitment than ExIMS. Investigators in exercise trials with other clinical groups have reported similar recruitment rates, such as wheelchair users (2.9/month) [11], breast cancer survivors (3.8/month) [10] and elderly stroke survivors (4.0/month) [23]. This suggests that our observed recruitment rate of 3.5 participants per month is a realistic target for future RCTs involving exercise for PwMS that require regular attendance.

### Response rates

The response rate from a potentially large pool of participants at MS clinics was low at 6.4 %. The reasons for this may be related to patients being ineligible (changing to new medication, experiencing a relapse, new patient, other neurological condition), consultants too busy to recruit during clinic time and/or patients disinterested in the study. As might be expected, response rates to personalised consultant study invitation letters were higher (42.8 %), as this strategy was targeted much more towards eligible individuals. However, this still leaves nearly 60 % of potential participants who did not respond to the invitation. As suggested by Daley et al. [10], it is possible that non-responders were either deterred by the demanding nature of exercise trials or were already engaged in regular physical activity. The latter seems less likely, owing to the lower physical activity rates reported in PwMS [24].

### Randomisation yields/accrual rates

The trial recruited 60 % of the 120 participants from the MS outpatient clinic, with 29.2 % recruited via consultant mail-out and 10.8 % via trial awareness strategies. However, the randomisation yield (number recruited/number interested) was similar for both the MS clinic and consultant mail-outs (33.2 % and 31.0 %, respectively), suggesting that both methods are useful in attaining recruitment targets. Values reported in the exercise literature are varied, with an exercise trial for wheelchair users reporting a randomisation yield of 41.8 % [11] and an exercise trial with breast cancer survivors reporting yields of 13.3 % from consultant letters and 29.7 % from community strategies. In addition, a cognitive behavioural trial for PwMS had relatively low yields of 4.5 % for the MS clinic and 4.0 % from mail-outs [21]. Hence, our data suggest that PwMS are as interested as other clinical populations in participating in a supervised exercise trial and may be more interested in an exercise trial than other behavioural interventions with similar time constraints.

### Reasons for ineligibility

There were a number of reasons why people interested in the trial were ineligible to take part. The most common reason for ineligibility was already being too active to participate (69.2 %) owing to already being engaged in moderate structured exercise at least three times per week for at least 30 minutes per session consistently for the last 6 months. This is consistent with reasons for non-eligibility reported in a similar exercise intervention with breast cancer survivors in which 55 % of those interested were ineligible owing to already being too active [10]. The number of potential participants screened out for already being too active was much less (8.5 %) in a group of wheelchair users [11], suggesting that physical disability may impact heavily on current exercise levels. Our data suggest that despite the physical disabilities of MS, there are many people with mild to moderate levels of disability due to the condition who are managing to participate in moderate-intensity exercise over a prolonged period. However, data from the wheelchair exercise study [11] would suggest that PwMS who have higher levels of disability may be less physically active.

### Reasons for choosing not to participate

The reasons that eligible participants have given for choosing not to take part in exercise intervention studies have rarely been reported, but they can offer valuable insight into areas of trial design that may be improved to enhance recruitment. Many PwMS (66.3 %) did not specify why they had declined to take part. However, of those who did, the need to travel to the trial site, negative perceptions of exercise and loss of benefit worries were all factors that could potentially be overcome in future trials through design modifications and patient education.

### Recruitment time/cost

Recruitment is a time-consuming process, with researchers in some community-based trials reporting up to 10 h per participant to recruit [25]. The present study average was 2.9 h per participant. Study mail-outs were reported to be the most efficient recruitment method at only 0.6 h per participant. However, only 29.2 % of the study’s overall cohort recruited by this method, suggesting the importance of the more time-consuming method of recruitment through MS outpatient clinics. Although this method required 4.2 h per participant, it yielded 60 % of the study’s total cohort. In the present study, costs were based on a researcher doing all the recruitment, regardless of method; however, if recruitment at the clinic had incurred additional consultant time, costs for this method would have been much higher.

### Limitations

There was the potential for cross-contamination across recruitment pathways, as participants may have been reached by more than one method. For example, PwMS may have seen trial awareness information before attending an appointment at the MS clinic, which may have made them more likely to be recruit by this method. This could be improved in future studies by asking participants if they have been made aware of the study by any other means. In addition, it was not a requirement of the study for individuals to provide reasons for declining to take part in the study. It would be useful to include methods for collecting these data so that strategies could be developed to increase recruitment yield and hence decrease recruitment costs.

## Conclusions

Achievement of predetermined recruitment targets is a critical factor influencing the success of RCTs. Well-designed feasibility work and a combination of recruitment methods can help to ensure that a trial is appropriately designed to reach targets. Although consultant mail-outs were shown to be the most cost-effective recruitment strategy, this method alone may well be insufficient to meet recruitment targets in time-limited RCTs. In this study, we report, for the first time to our knowledge, the pros and cons of different recruitment methods in RCTs involving exercise for PwMS. On the basis of our results, we recommend a combination of methods to meet recruitment targets. The results provide novel insights into challenges of trial recruitment in this context and can be used to inform the design of future trials in this population; recruitment for other types of trials, such as drug trials, may be different.

## Additional file

Additional file 1:
**CONSORT checklist and flow diagram.** (DOCX 193 kb)

## Abbreviations

CONSORT: Consolidated Standards of Reporting Trials
EDSS: Expanded Disability Status Scale
ExIMS: Exercise Intervention for Multiple Sclerosis
MS: Multiple sclerosis
PwMS: People with multiple sclerosis
RCT: Randomised controlled trial
