# Supplementary material for: Participant recruitment into a randomised controlled trial of exercise therapy for people with multiple sclerosis
Source: Trials. 2015 Oct 15;16:468. doi: 10.1186/s13063-015-0996-3 (PMC4608102; doi:10.1186/s13063-015-0996-3)

**CONSORT: Checklist and Flow Diagram**

**Checklist:**

| **TITLE AND ABSTRACT** | |
| --- | --- |
| **1 a. Title - Identification as a randomised trial in the title.**  "Participant recruitment into a randomised controlled trial of exercise therapy for people with multiples sclerosis." | **✓** |
| **1 b. Abstract - Structured summary of trial design, methods, results, and conclusions**  See abstract in document | **✓** |
| **INTRODUCTION** | |
| **2 a. Background - Scientific background and explanation**  "One of the most difficult challenges in clinical trials is whether appropriate participants can be identified and consented quickly (Lindbald, Zingeser and Sismanyazici, 2011). Many trials either fail to reach recruitment targets or have to be extended (McDonald et al., 2006). This then either leads to an under-powered study, or an extension to the duration of the study often at additional cost, impacting on the time required to inform clinical practice and utilising funds that could have been used for other research (Treweek et al., 2011; McDonald et al., 2011). The implementation of an efficient and effective recruitment strategy for patients on clinical trials is critical if expensive delays and failures to meet predetermined targets are to be avoided (Probstfield and Frye, 2011). | **✓** |
| **2 b. Objectives - Specific objectives or hypotheses**  "The main purpose of this report is to provide a detailed outline of the recruitment methods, rates and estimated cost to help inform future research of this type. In addition, we aim to determine which recruitment method provided the highest yield of participants and the lowest cost per participant." | **✓** |

| **METHODS** | |
| --- | --- |
| **3 a. Trial Design - Description of trial design ( such as parallel, factorial) including allocation ratio**  Only a brief description is included in this recruitment paper as a more detailed protocol and main trial paper are published elsewhere, as referenced in the methods section;  "A brief description of the trial design is reported here as detailed protocol and outcomes papers for this study have been published elsewhere (Saxton, et al., 2013; Carter et al., 2014; Tosh et al., 2014)." | **✓** |
| **3 b. Changes to trial design - Important changes to methods after trial commencement (such as eligibility criteria), with reasons**  No changes were made and therefore none needed to be reported | **✓** |
| **4 a. Participants - Eligibility criteria for participants**  "Regardless of the recruitment method used, all participants were screened by a consultant neurologist prior to entering the trial. Participants were included if they; had a clinical diagnosis of MS using the McDonald diagnostic criteria for MS (Polman et al 2011); had an Expanded Disability Status Score (EDSS) (Kurtze, 1983) between 1.0 and 6.5; aged 18-65 years; were stable on disease modifying treatment for ≥ three months prior to recruitment; were clinically stable (had not experienced a relapse in at least four weeks); were physically able to participate in exercise three times per week and were able to provide written informed consent. Exclusion criteria were; failure to meet any of the inclusion criteria; experiencing illness that would be a contra indicator to exercise; living further than 20 miles from the trial centre; unwilling to be randomised to either group; already engaged in moderate structured exercise ≥ three times per week for ≥ 30 min per session consistently for the last six months. Participants who were initially screened out due to either having changed their drug treatment in the last three months or having had a relapse in the previous four weeks were re-assessed following the required lapse of time and recruited if the eligibility criteria were then met. " | **✓** |
| **4 b. Study Settings - Settings and locations where data were collected**  Again as mentioned in section 3 a. more detailed information regarding the trial setting is referenced, as this is included in the protocol and mains study papers (Saxton et al., 2013; Carter et al., 2014). published elsewhere. However, detail is provided regarding the setting for recruitment;  "at MS outpatient clinics"  "took place at the Royal Hallamshire Hospital"  "at clinics, therapy centres and regional MS Societies, presentations and attendance at regional MS Society events and to local MS physiotherapy teams" | **✓** |
| **5. Interventions - The interventions for each group with sufficient details to allow replication, including how and when they were actually administered**  As this was a recruitment paper, it contains detail around the recruitment methods;  "MS outpatient clinics took place at the Royal Hallamshire Hospital, Sheffield on a weekly basis. The project's lead consultant (BS) and two other neurology consultants assisted with identifying potentially eligible and interested participants. Each consultant saw approximately 13 patients per clinic (10 follow-ups and three new patients) over a 3.5-hour period. A trial researcher attended all clinics, enabling any participants identified to speak with them about the trial, ask any questions and confirm eligibility. If interested, participants were booked in for a familiarisation session at the trial site."  Some information was provided regarding the actual intervention  " Participants were randomized to a 12-week pragmatic exercise intervention (2 x supervised and 1 x home-based session per week for 6-weeks followed by 1 x supervised and 2 x home-based sessions per week for 6-weeks, plus usual care) or usual care alone. "  More detailed information regarding the intervention itself is published elsewhere in the protocol paper (Saxton et al., 2013) for this study. | **✓** |
| **6 a. Outcomes - Completely defined pre-specified primary and secondary outcome measures, including how and when they were assessed**  The primary and secondary outcome measures for the main trial were reported, with further detail available in the protocol paper (Saxton et al., 2013);  "The primary outcome was self-reported exercise behaviour at 3-months using the Godin Leisure Time Exercise Questionnaire (GLTEQ) (Godin et al., 1985). In addition accelerometry was used to provide an objective measure of daily activity and step count (Actigraph GT2M accelerometer, Actigraph, LLC, FL, USA). Secondary outcome measures included fatigue, health related quality of life, functional ability and neurological impairment. Outcomes were assessed at baseline, immediately post intervention (3 months) and 6 months post intervention (9 months)." | **✓** |
| **6 b. Changes to outcomes - Any changes to trial outcomes after the trial commenced, with reasons**  No changes required. | **✓** |
| **7 a. Sample size - How sample size was determined**  "Power calculations indicated that we would need 100 PwMS to complete the trial. This alongside the retention rates observed in our feasibility study of 87% immediately following the intervention and 80% at 3-months (Carter et al., 2013), lead to a recruitment target of 120 PwMS (60 in each group)."  More detail is outlined in the studies protocol paper (Saxton et al., 2013) | **✓** |
| **7 b. Interim analyses and stopping guidelines**  Not applicable | **✓** |
| **8 a. Randomisation sequence generation - Method used to generate random allocation sequence**  This is detailed in the studies protocol (Saxton et al., 2013) paper referenced in the article | **✓** |
| **8 b. Randomisation: Type - Type of randomisation; details of any restriction (such as blocking and block size)**  This is detailed in the studies protocol paper (Saxton et al., 2013) referenced in the article | **✓** |
| **9. Randomisation: allocation concealment mechanism - Mechanism used to implement the random allocation sequence (such as sequentially numbered containers), describing any steps taken to conceal the sequence until interventions were assigned**  This is described in the protocol and main trial papers (Saxton et al., 2013; Carter et al., 2014). referenced in this article. | **✓** |
| **10. Randomisation: implementation - Who generated the allocation sequence, who enrolled participants, and who assigned participants to interventions**  This is described in the protocol and main trial papers (Saxton et al., 2013; Carter et al., 2014). referenced in this article. | **✓** |
| **11a. Blinding - If done, who was blinded after assignment to interventions (for example, participants, care providers, those assessing outcomes) and how**  This is described in the protocol (Saxton et al., 2013) and main trial (Carter et al., 2014) papers referenced in this article. | **✓** |
| **11b. Similarity of interventions - If relevant, description of the similarity of interventions**  Not applicable | **✓** |
| **12a. Statistical methods - Statistical methods used to compare groups for primary and secondary outcomes**  This is a recruitment paper these statistics are included in the protocol and main trial paper published elsewhere (Saxton et al., 2013; Carter et al., 2014).  However data analysis methods for recruitment were included in this paper;  "Data analysis: Participant recruitment rates were calculated as the average number of participants recruited per month over the duration of the recruitment period. Response rates were reported as percentage interested and percentage recruited. Recruitment yields were calculated as total recruited divided by the number of interested participants. Recruitment time was estimated based on time taken to ascertain interest and eligibility in the study and does not include any other time taken to carry out familiarisation visits and consent as this was the same for all recruitment methods. The time cost of each method is calculated per participant recruited, based on the average salary cost per hour of the trial researcher." | **✓** |
| **12b. Additional analyses - Methods for additional analyses, such as subgroup analyses and adjusted analyses**  See 12 a | **✓** |

| **RESULTS** | |
| --- | --- |
| **13a. Participant Flow - For each group, the numbers of participants who were randomly assigned, received intended treatment, and were analysed for the primary outcome**  As this is a recruitment paper participant flow is reported up to randomisation. Details for participant flow in the trial are included in the main study paper (Carter et al., 2014). | **✓** |
| **13b. Losses and exclusions - For each group, losses and exclusions after randomisation, together with reasons**  Not applicable due to recruitment nature of this paper. Details on this are included in the main trial paper (Carter et al., 2014). | **✓** |
| **14a. Recruitment - Dates defining the periods of recruitment and follow-up**  " The original recruitment period was planned to take place over a period of 24 months. This was extended to a period of 34 months (February 2009 to November 2011), due to lower than expected recruitment rate of 3.5 ± 0.32 (mean ± 95% CI) participants per month (See Fig. 2)." | **✓** |
| **14b. Reason for stopped trial - Why the trial ended or was stopped**  Not applicable | **✓** |
| **15. Baseline Data- A table showing baseline demographic and clinical characteristics for each group**  This is not applicable as this paper reports the recruitment for the project and therefore covers the period prior to randomisation into groups. | **✓** |
| **16. Numbers analysed - For each group, number of participants (denominator) included in each analysis and whether the analysis was by original assigned groups**  See answer to qu. 15. | **✓** |
| **17a. Outcomes and estimation - For each primary and secondary outcome, results for each group, and the estimated effect size and its precision (such as 95% confidence interval)**  Due to the recruitment nature of this paper, this data is not required. However it is included in the main trial paper (Carter et al., 2014). | **✓** |
| **17b. Binary outcomes- For binary outcomes, presentation of both absolute and relative effect sizes is recommended**  See answer to qu. 7a. | **✓** |
| **18. Ancillary analyses - Results of any other analyses performed, including subgroup analyses and adjusted analyses, distinguishing pre-specified from exploratory**  See answer to qu. 7 a. | **✓** |
| **19. Harms- All important harms or unintended effects in each group**  This paper follows the recruitment phase and therefore does not cover the intervention reported elsewhere (Carter et al., 2014). | **✓** |
| **DISCUSSION** | |
| **20. Limitations- Trial limitations, addressing sources of potential bias, imprecision, and, if relevant, multiplicity of analyses**  Trial limitations are included in the main trial paper (Carter et al. 2014). However, limitations for recruitment are reported here;  "Limitations - There was the potential for cross-contamination across recruitment pathways, as participants may have been reached by more than one method (for example PwMS may have seen trial awareness information, before attending an appointment at the MS clinic, which may have made them more likely to recruit from this method). This could be improved in future studies by asking participants if they have been made aware of the study by any other means. In addition, it was not a requirement of the study for individuals to provide reasons for declining to take part in the study/ it would be useful to include methods for collecting this data so that strategies can be developed to increase recruitment yield and hence decrease recruitment costs." | **✓** |
| **21. Generalisability - Generalisability (external validity, applicability) of the trial findings**  Some information provided regarding recruitment findings, main trial findings are reported elsewhere (Carter et al., 2014);  "The results provide novel insights into challenges of trial recruitment in this context and can be used to inform the design of future trials in this population; recruitment for other types of trial such as drugs trials may be different." | **✓** |
| **22. Interpretation- interpretation consistent with results, balancing benefits and harms, and considering other relevant evidence**  " This is consistent with reasons for non-eligibility reported in a similar exercise intervention with breast cancer survivors where 55% of those interested were ineligible due to being already too active (Daley et al., 2007). The number of potential participants screened out through being already too active was much less (8.5%) in a group of wheelchair users (Nary et al., 2011), suggesting that physical disability may impact heavily on current exercise levels." | **✓** |
| **OTHER INFORMATION** | |
| **23. Registration - Registration number and name of trial registry**  "International Standard Randomised Controlled Trial Number: ISRCTN41541516; Date registered: 05/02/2009" | **✓** |
| **24. Protocol - Where the full trial protocol can be accessed, if available**  Saxton JM, Carter A, Daley AJ, Snowdon N, Woodroofe MN, Petty J, Roalfe A, Tosh J, Sharrack B (2013) Pragmatic exercise intervention for people with multiple sclerosis (ExIMS Trial): study protocol for a randomised controlled trial. Contemporary Clinical Trials, 34: 205-11 | **✓** |
| **25. Funding - Sources of funding and other support (such as supply of drugs), role of funders**  " This work was supported by a grant from the MS Society (Grant number 888/08), with additional support for recruitment from consultants Dr S Price and Dr S Howell from Sheffield Teaching Hospitals NHS Trust." | **✓** |

**Flow diagram:**

A flow diagram for recruitment was included in this paper (figure 1.)

A full CONSORT diagram is included in the main trial paper (Carter et al., 2014) and has been included below.


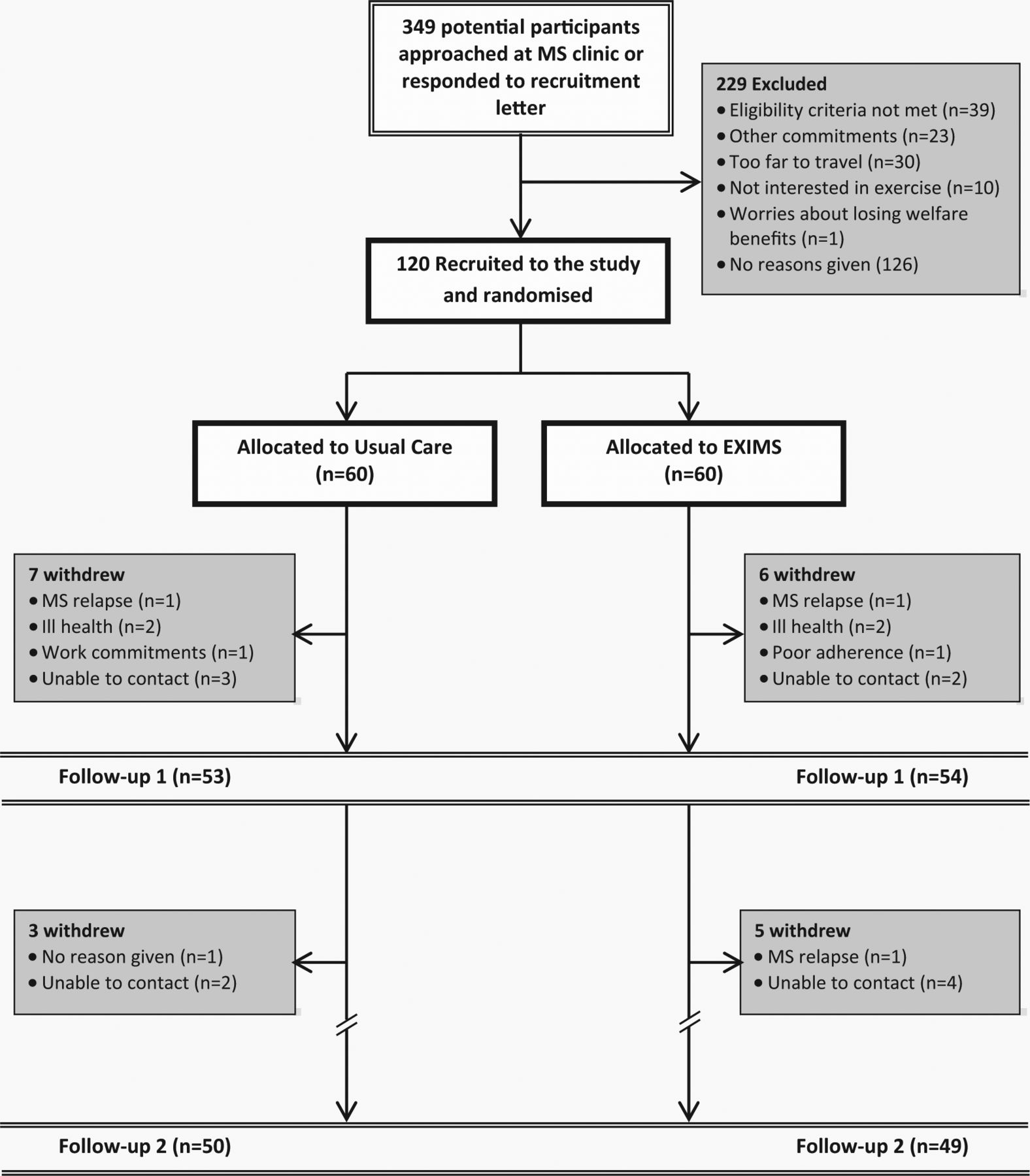

Supplement: Additional file 1: — CONSORT checklist and flow diagram. (DOCX 193 kb) [file 13063_2015_996_MOESM1_ESM.docx]
